# RNase H genes cause distinct impacts on RNA:DNA hybrid formation and mutagenesis genome wide

**DOI:** 10.1126/sciadv.adi5945

**Published:** 2023-07-26

**Authors:** Jeremy W. Schroeder, Rebecca L. Hurto, Justin R. Randall, Katherine J. Wozniak, Taylor A. Timko, Taylor M. Nye, Jue D. Wang, Lydia Freddolino, Lyle A. Simmons

**Affiliations:** ^1^ Department of Biological Chemistry, University of Michigan Medical School, Ann Arbor, MI 48109, USA.; ^2^ Department of Molecular, Cellular, and Developmental Biology, University of Michigan, Ann Arbor, MI 48109, USA.; ^3^ Department of Bacteriology, University of Wisconsin - Madison, Madison, WI 53706, USA.; ^4^ Department of Molecular Virology and Microbiology, Baylor College of Medicine, Room 743E, Houston, TX 77030, USA.

## Abstract

RNA:DNA hybrids compromise replication fork progression and genome integrity in all cells. The overall impacts of naturally occurring RNA:DNA hybrids on genome integrity, and the relative contributions of ribonucleases H to mitigating the negative effects of hybrids, remain unknown. Here, we investigate the contributions of RNases HII (RnhB) and HIII (RnhC) to hybrid removal, DNA replication, and mutagenesis genome wide. Deletion of either *rnhB* or *rnhC* triggers RNA:DNA hybrid accumulation but with distinct patterns of mutagenesis and hybrid accumulation. Across all cells, hybrids accumulate strongly in noncoding RNAs and 5′-UTRs of coding sequences. For Δ*rnhB*, hybrids accumulate preferentially in untranslated regions and early in coding sequences. We show that hybrid accumulation is particularly sensitive to gene expression in Δ*rnhC* cells. DNA replication in Δ*rnhC* cells is disrupted, leading to transversions and structural variation. Our results resolve the outstanding question of how hybrids in native genomic contexts cause mutagenesis and shape genome organization.

## Teaser

Bacterial RNase H enzymes impact RNA:DNA hybrid accumulation resulting in distinct mutagenetic changes in the genome.

## INTRODUCTION

RNA:DNA hybrids (referred to herein as RDHs) form through a variety of essential processes such as transcription and DNA replication, giving rise to several distinct forms of RDHs in vivo. RDHs include single ribonucleotide misincorporation errors by replicative DNA polymerases, primers for Okazaki fragments during lagging strand replication, and R loops formed when a single strand of RNA anneals to complementary DNA, displacing a strand of DNA in the process [reviewed in (*1*–*3*)]. RDHs are problematic for several potential reasons: First, they are more stably paired than double-stranded DNA, making their removal by helicases difficult; second, RNA is much more susceptible than DNA to breaks due to the reactive 2′-hydroxyl group on the ribose sugar; last, R loops can pose barriers to DNA replication (*4*–*8*). The relative contribution of these biological and chemical properties of RDHs to genome instability is unclear. However, what is clear is that in organisms ranging from bacteria to humans, RDHs promote the accumulation of mutations that threaten genome integrity, cause double-strand breaks, and result in activation of the innate immune response in human cells (*6*, *9*–*14*).

Because RDHs can be costly to cells, mechanisms exist to remove them. For example, in bacteria, there are three types of ribonuclease (RNase) H enzymes capable of digesting the RNA strand of an RDH: HI, HII, and HIII (*15*). Most bacteria, including *Escherichia coli*, encode RNases HI and HII. However, an important subset of bacteria, including the Gram-positive bacterium *Bacillus subtilis*, contains RNases HII and HIII (*16*, *17*). All three types of RNase H enzymes can incise ribonucleotides covalently embedded in DNA (*18*, *19*). In contrast, only RNases HI and HIII incise substrates lacking a covalent RNA-DNA junction, suggesting that these enzymes cleave R loops in vivo (*18*, *19*).

The mechanisms through which RNase H enzymes maintain genome stability are multifaceted. RNase HII may reduce mutagenesis by enabling relatively error-free replacement of ribonucleotides misinserted by DNA polymerases (*10*, *20*, *21*). In *E. coli*, RNase HI suppresses double-strand break formation at sites of codirectional conflicts between DNA replication and transcription (*22*). Head-on replication-transcription conflict is especially deleterious (*7*, *22*–*26*), and RNase HIII may reduce R loop accumulation and mutation rates in head-on genes, potentially by helping to avoid replication fork reversal at head-on conflict sites (*7*, *27*). Assigning relative importance to the contributions and interplay of RDHs, replication-transcription conflict direction, gene expression level, R loop stability, gene location, and sequence context to mutagenesis has been difficult, in part because of the sheer number of biological inputs, and in part owing to the current lack of a highly specific, reproducible method for detecting R loops.

To understand the impact of RDHs such as R loops on genome integrity, it is necessary to have a highly specific method for RDH detection (*28*) while also accounting for confounding biological variables such as gene expression and transcription direction relative to DNA replication. We therefore used a recently developed, high-accuracy method for RDH detection and measured genome-wide RDH levels, alongside measurement of gene expression via RNA sequencing (RNA-seq), in *B. subtilis* cells lacking either RNase HII (*rnhB*) or HIII (*rnhC*). To relate these findings to mutagenesis, we integrated our gene expression and RDH abundance measurements with mutation accumulation (MA) line data using a statistical model that accounted for the diverse biological inputs affecting genome stability. We show that in the absence of RnhC, R loops accumulate in regions of high expression and cause severe genome instability due to replication stress at a specific locus with pervasive RDH presence near the chromosomal replication terminus. By contrast, in cells lacking RnhB, RDHs accumulate preferentially in untranslated regions (UTRs) and early in coding sequences. Our integrative approach reveals that R loops, regardless of gene orientation, promote accumulation of insertions and deletions in coding sequences in wild-type cells. However, in nearly all cases, the selective pressures exerted by RDH formation appear to have minimized the potential for problematic conflicts in naturally evolved genes. Our combined analyses provide a genome-wide assessment for how transcription, DNA replication, and R loops contribute to mutation rate and replication fork perturbations throughout a bacterial chromosome.

## RESULTS

### Loss of RnhB or RnhC results in global changes in RDH distributions

**Fig. 1.**
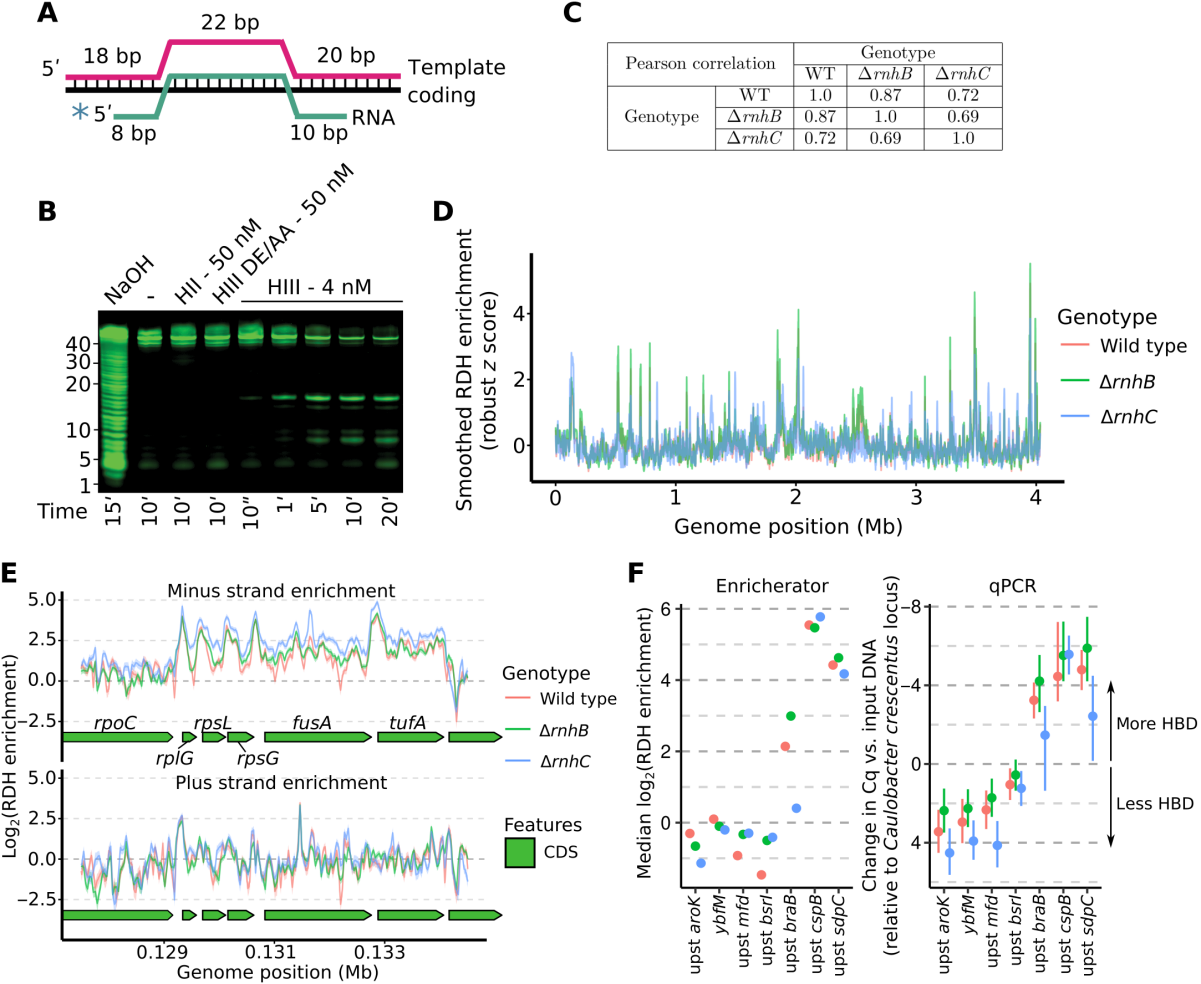
Loss of *rnhB* and/or *rnhC* causes widespread increases in RDH formation. (**A**) Diagram of the three oligonucleotides used to generate the R loop substrate for in vitro cleavage reactions. (**B**) Urea-PAGE of the R loop treated with 50 nM RnhB (HII), 50 nM catalytically inactive RnhC (HIII DE/AA), or 4 nM RnhC (HIII) at the indicated time points. (**C**) Pearson correlation coefficients for genome-wide comparison of RDH enrichment scores across genotypes. (**D**) Overview of RDH signal, smoothed using a 10-kb sliding median across the genome for each genotype. (**E**) Strand-specific RDH enrichment at a specific region of the genome. Lines represent the mean, and shaded intervals represent the 90% quantile interval of 500 samples from the approximate posterior distribution. Note that the shaded intervals are often narrow and are thus not always visible under the mean line. Estimates were attained using data from two biological replicates. (**F**) Comparison of locus-specific enrichments inferred by Enricherator (left) and as determined by qPCR (right). For the qPCR plot, points represent the mean of the samples from each posterior distribution and bars represent 95% highest posterior density intervals. Two biological replicates were used, and two to three technical replicates of PCR reactions were performed (see data file S1 for original data). WT, wild type.

We began by testing the ability of *B. subtilis* RnhB (HII) and RnhC (HIII) to incise an R loop substrate in vitro (Fig. 1, A and B). RnhB was not active against the R loop, while RnhC efficiently cleaved the RNA strand of the R loop substrate (Fig. 1, A and B, and fig. S1). Given the differing specificities of RnhB and RnhC enzymes in vitro, we expected that loss of *rnhB* and/or *rnhC* would influence the genome-wide abundance and distribution of RDHs in distinct ways. We used an epitope-tagged hybrid-binding domain (HBD) from human RNase H1 (*29*) to isolate RDHs with four or more ribonucleotides from wild-type (PY79), ∆*rnhB*, and ∆*rnhC* cells followed by high-throughput sequencing of the input and RDH-enriched DNA. We quantified genome-wide RDH enrichment using a tool developed for this work called Enricherator (see Supplementary Methods for details and table S1).

RDH enrichment scores varied over the genome similarly across genotypes, with Pearson correlation coefficients for any two genotypes between 0.69 and 0.87 (Fig. 1, C and D). Wild-type and Δ*rnhB* RDH enrichments were highly correlated, indicating a similar distribution (Pearson correlation = 0.87), whereas Δ*rnhC* showed substantial differences in overall RDH density from Δ*rnhB* and WT. In general, highly transcribed regions were enriched for RDHs, especially in Δ*rnhC* cells (Fig. 1E). We sought to compare RDH accumulation across genotypes, and because our sequencing approach can only yield relative levels of enrichment within a given genotype, we performed quantitative polymerase chain reaction (qPCR) of several genomic regions informed by our sequencing-based enrichments. To ensure we were comparing RDH enrichments to a known, unchanging reference locus, we spiked in an equal amount of *Caulobacter crescentus* cell pellet to each sample before lysing cells for input DNA and HBD pull-down procedures. HBD enrichment as determined by qPCR was defined in terms of ΔΔCq. Here, ΔΔCq is the difference in Cq between HBD pull-down versus input DNA at a given *B. subtilis* locus compared to the difference in Cq between HBD pull-down versus input DNA at a single *C. crescentus* transfer RNA (tRNA)^Thr^ locus (reflecting a region with known and robust RDH formation). Comparing our genome-wide estimates from Enricherator and our targeted qPCR approach, differences in RDH pull-down efficiency across genotypes and loci were in excellent agreement between the two methods (Fig. 1F). Notably, most loci tested by qPCR displayed slightly lower RDH pull-down efficiencies for Δ*rnhC* cells than for wild-type and Δ*rnhB* cells. Although based on the known biochemistry of RnhC and RnhB, we initially expected the opposite to be the case (*19*); Δ*rnhC* cells are sensitive to several types of stress [see below and (*7*)], and we suggest that the absence of RnhC may globally reduce transcription due to the stress induced by loss of RnhC. Globally reduced transcription in Δ*rnhC* cells would in turn cause most loci to display lower RDHs per unit genome, such that only the loci with very high expression display higher RDHs in Δ*rnhC* cells. Note that this cross-strain quantitation of RDH abundances can only be performed in the presence of a uniform spike-in reference.

### RDHs are enriched at highly expressed genes, especially in cells lacking RnhC

**Fig. 2.**
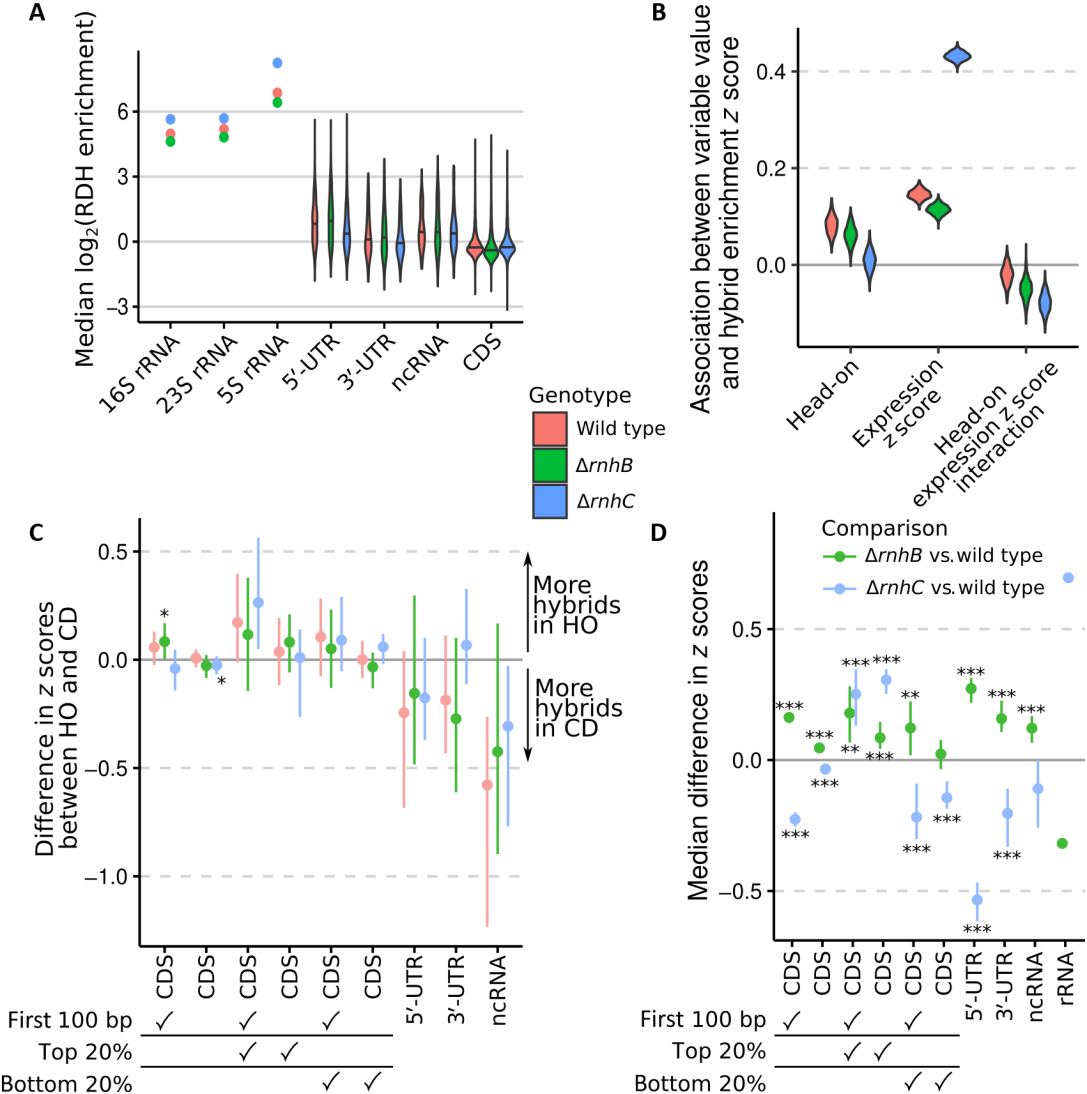
Loss of *rnhB* and/or *rnhC* causes changes to RDH signals for codirectional and head-on genes. RDH enrichment for each feature was the median robust *z* score for RDH enrichment within the feature. False discovery rate (FDR) indicators for (C) and (D): *, 0.01 ≤ FDR < 0.05; **, 0.001 ≤ FDR < 0.01; ***, FDR < 0.001. Wilcoxon rank-sum tests were performed. (**A**) Distributions of RDH enrichment scores for each feature on the *x* axis. For rRNA, there is one point per genotype because the reference genome contained a single consensus sequence for each rRNA, with the 10 chromosomal copies masked. Therefore, for each rRNA, a single enrichment score exists, whereas for other feature types, we display the distributions of enrichment scores. Horizontal lines across the violin plots represent the median value for each group. (**B**) Sampled posterior distributions are displayed for a model regressing RDH enrichment *z* score, aggregated by CDS against gene orientation relative to DNA replication, gene expression’s *z* score, and an interaction term between gene orientation and expression (“Bayesian inference of genome-wide RNA:DNA hybrid enrichment scores” section in Supplementary Materials and Methods). (**C**) Difference in head-on (HO) versus codirectional (CD) feature RDH enrichment scores (points) is displayed with the 95% credible interval (CI; bars). Check marks under the *x*-axis labels indicate whether filters for position within CDSs or gene expression were applied for a given set of comparisons. (**D**) RDH enrichment *z* scores were compared across genotypes, paired by genomic feature. The resulting median feature-wise difference in RDH enrichment is plotted (points) along with the 95% CI (bars). Because of the low number of rRNA in our reference genome, we did not estimate confidence intervals or perform hypothesis tests for rRNA. Thus, the rRNA comparisons are plotted as points to indicate that they represent point estimates of the differences. All estimates were attained using data from two biological replicates. ncRNA, noncoding RNA.

To interpret the genome-wide patterns of RDH abundance observed in our experiments, we separately analyzed the distributions of RDH signals across several classes of genomic features (e.g., open reading frames, 3′-UTRs, etc.). Across all considered strains, ribosomal RNA (rRNA) loci had the highest levels of RDH accumulation, with 5′-UTRs having overall higher RDH accumulation than other features (Fig. 2A). Loss of *rnhC* further exacerbated RDH accumulation at rRNA loci.

Active expression of protein coding genes and the direction of gene transcription relative to that of DNA replication (head-on versus codirectional) have been correlated with changes in R loop enrichment (*7*, *30*). We therefore expected that the most actively expressed genes should have increased RDH enrichment and that the distribution of RDH scores for head-on and codirectional genes and for the most actively expressed genes may change in the absence of *rnhB* or *rnhC*. We fit a statistical model to regress RDH enrichment scores for coding sequences (CDSs) against CDS expression, direction of transcription relative to DNA replication (head-on or codirectional), and the interaction between CDS orientation and expression. For all strains, increased CDS expression was associated with higher RDH enrichment scores, but this association was much more pronounced for Δ*rnhC* cells than for either wild type or Δ*rnhB* (Fig. 2B). For wild-type and Δ*rnhB* cells, head-on orientation of a CDS was associated with a small increase in RDH enrichment *z* score, but increased gene expression in head-on genes had a lower association with RDH abundance (Fig. 2B), suggesting that existing highly expressed head-on genes in *B. subtilis* may have evolved to have lower R loops than equivalent expression levels in codirectional genes would predict. Further, we find no association between R loops and loss of *rnhC* in head-on–expressed genes.

Next, we explored the association between gene orientation, expression, and RDH enrichment by separating genomic features into categories and calculating the difference in RDH enrichment *z* scores between head-on and codirectional features. The first 100 base pair (bp) of head-on CDSs, especially for highly expressed CDSs when *rnhB* was absent, displayed slightly higher RDH enrichment scores than codirectional CDSs (Fig. 2C). However, when the entire length of CDSs was considered, head-on and codirectional CDSs showed similar RDH accumulation, regardless of genotype. For noncoding RNA other than rRNA and tRNA, head-on transcription is associated with lower RDH enrichments (Fig. 2C).

Comparing RDH enrichment *z* scores across genotype, we observed higher *z* scores in ∆*rnhB* cells (relative to wild type) across nearly all considered genomic features aside from rRNAs. Our finding is consistent with generally pervasive but less transcription-dependent RDHs in the absence of RnhB, because for highly expressed genes RDHs accumulate less in ∆*rnhB* than in ∆*rnhC* (Fig. 2, B and D). Further underscoring the strong relationship between transcription and R loop formation in ∆*rnhC* cells (Fig. 2B), RDH *z* scores for ∆*rnhC* cells were higher than wild type in the 20% most highly expressed CDSs but were otherwise lower than wild type when considering all CDSs, CDSs with low expression, and UTRs (Fig. 2D). We conclude that while all cells accumulate RDHs strongly at highly expressed genes, RDH accumulation in the ∆*rnhC* genotype is more strongly affected by transcription, while RDH accumulation in *∆rnhB* increases more evenly throughout the genome.

### Replication stress occurs at long head-on operons near the chromosomal terminus in ∆*rnhC cells*

As loss of *rnhB* or *rnhC* cause different patterns of RDH accumulation and increases in R loop formation are associated with genomic instability, we expected not only that ∆*rnhB* and ∆*rnhC* cells would have higher mutation rates than wild-type cells but also that the types and frequencies of the mutations would differ in each genetic background. To understand the genome-wide contribution of RDHs to mutagenesis, we performed MA experiments using Δ*rnhC* cells and compared mutagenesis in Δ*rnhC* to previously published MA line results from wild type and ∆*rnhB* (*10*, *31*).

**Fig. 3.**
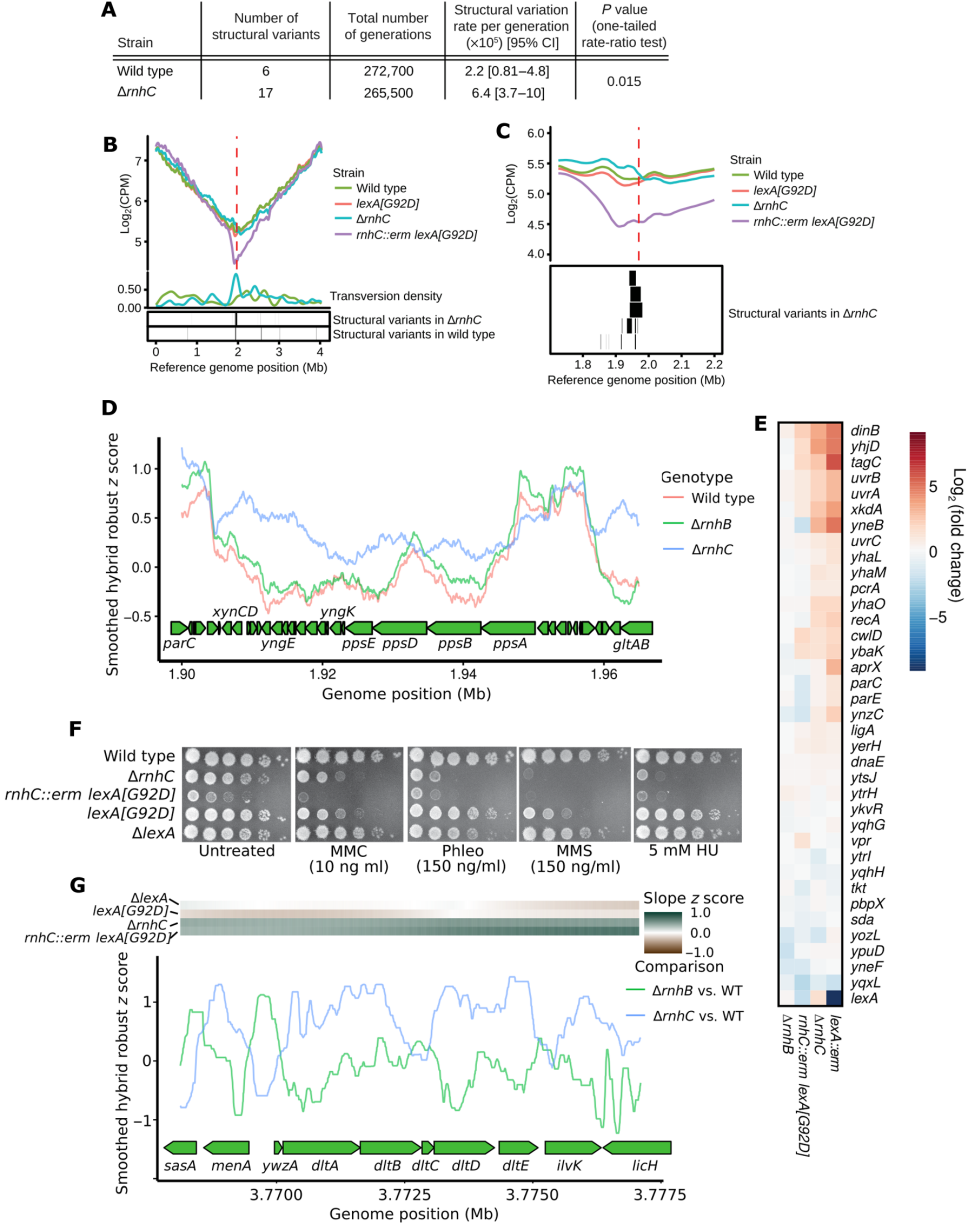
RnhC suppresses replication stress and genome instability. (**A**) Table showing structural variants from wild-type and Δ*rnhC* MA lines. (**B**) Log_2_-transformed sequencing read counts per million reads mapped (CPM) plotted versus genome position for wild type, *lexA[G92D]*, Δ*rnhC*, and *rnhC::erm lexA[G92D]*. Density of transversions is plotted (see Materials and Methods) below versus genome position for Δ*rnhC* and wild type. The red dashed line indicates the replication terminus. (**C**) Top: A view of the data plotted in (B), zoomed in on the region near the replication terminus (red dashed line). Bottom: Structural variants detected in Δ*rnhC* MA lines are plotted as boxes to indicate their genome position. (**D**) For each strain, RDH enrichment scores from Enricherator were converted to robust *z* scores, smoothed using a 10-kb rolling median, and plotted versus genome position near the *pps* operon. (**E**) Shown is a heatmap of gene expression for the SOS response in the indicated genetic backgrounds relative to wild type. Three biological replicates were performed for each genotype. (**F**) Spot titer assays of various RnhC and LexA-deficient strains serial diluted 10^−5^ and plated on LB with the chemical stress indicated in the figure. (**G**) Contrasts for each of Δ*rnhB* and Δ*rnhC* relative to wild type were prepared using Enricherator scores. Contrasts were converted to robust *z* scores, smoothed using a 500-bp rolling median, and plotted at the *dlt* operon. The heatmap above the plot shows the *z* score of the slope of the smoothed DNA sequencing coverage plotted in (B). Dark green scores indicate decreased rate of DNA replication progression.

For Δ*rnhC* cells, we completed 72 MA lines representing a total of ≈265,500 generations surpassed in the pooled lines. Among the genomic variations we detected in MA line data, we found that structural variants and transversions were particularly pronounced in Δ*rnhC* lines (Fig. 3A and data file S2). The total number of structural variants detected in Δ*rnhC* was 17 compared to the 6 identified in wild-type lines, resulting in a structural variation rate per generation of 6.4 × 10^−5^ for Δ*rnhC* compared to 2.2 × 10^−5^ for wild type (*P* = 0.015, one-tailed rate-ratio test; Fig. 3A). Structural variants clustered near the replication terminus in Δ*rnhC* lines (Fig. 3, B and C). We reasoned that because mutations clustered near the terminus of Δ*rnhC* cells, DNA replication fork progression could be impaired in this region of the chromosome. To test this, we monitored DNA replication status genome wide by performing whole-genome sequencing on DNA from exponentially growing cells. The slope of the genome sequencing coverage profiles served as a proxy for replication status in each strain, where a shallow slope was indicative of rapid replication fork progression, and a steep slope indicated replication proceeded with difficulty (*25*). For our genomic analysis of replication status, we sequenced DNA from wild-type, Δ*rnhC*, *lexA[G92D]*, and *rnhC::erm lexA[G92D]* cells. Cells harboring *lexA[G92D]* encode a noncleavable LexA protein and are impaired for induction of the SOS response. We found that the genome-wide replication profiles were similar for wild-type and *lexA[G92D]* cells, as indicated by similar slopes of genome sequencing coverage in each strain (Fig. 3, B and C). However, in cells both lacking *rnhC* and impaired for activation of the SOS response (*rnhC::erm lexA[G92D]*), we found a steep drop in sequencing coverage near the terminus region of the genome where several head-on genes reside, including *yngEFG*, *yngIJ*, and *ppsABDE* (Fig. 3, B and C). The drop in sequencing coverage indicates that DNA replication was substantially impaired as it approached the *ppsABDE* locus in the absence of *rnhC*. Replication stress in the *yng* and *pps* operons coincided with pervasive RDH increases in Δ*rnhC* relative to wild-type cells in these head-on genes (Fig. 3D), suggesting that DNA replication may be impaired at this locus due to pervasive R loop accumulation.

Inspection of the replication profiles suggests that the SOS response is induced in Δ*rnhC* cells. Using RNA-seq (Fig. 3E) and single-cell reporters (fig. S3A), we found that Δ*rnhC* cells were constitutively induced for the SOS response. RNA-seq results for the LexA regulon in Δ*rnhC* closely mirrored the Δ*lexA* (constitutive SOS) expression profile, whereas Δ*rnhB* cells did not show SOS induction (Fig. 3E). Further, we show that Δ*rnhC* cells were sensitive to hydroxyurea (HU) and a variety of DNA damaging agents when compared with wild-type cells, sensitivities that were exacerbated by concomitant loss of SOS induction (Fig. 3F). Our findings that loss of *rnhC* leads to SOS induction and sensitivity to DNA damage support and extend previous findings underscoring the conclusion that Δ*rnhC* cells are severely compromised for genome integrity (*7*, *19*, *20*). We conclude that SOS induction is critical for survival and proliferation of cells lacking *rnhC*, and diversion of the SOS regulon in replication stressed-Δ*rnhC* cells leads to their poor survival on DNA damaging agents. Further, we suggest that in the absence of *rnhC*, increased mutagenesis near the terminus of replication is caused by replication stress due to defects in R loop removal from the *yng* and *pps* operons in that region, followed by SOS induction.

In addition to replication problems near the *pps* operon, we detected a second site, the *dltABCDE* operon, where DNA replication proceeded slowly in cells lacking *rnhC* (Fig. 3G, top). The *dlt* operon is head-on and highly expressed, with the mean expression *z* score of the genes in the operon ranging from 1.5 to 1.7 in wild-type cells. Loss of *rnhC* caused increased and pervasive RDH accumulation in the *dlt* operon (Fig. 3G). Therefore, replication stress at the *dltABCDE* locus is likely the result of head-on replication-transcription conflict stabilized by R loops. However, unlike the *ppsABDE* operon, *dltABCDE* is distal to the terminus and did not show any changes in mutation or structural variation rates, indicating that high expression, R loop stabilization, and head-on orientation are not sufficient to increase rates of transversions and genomic rearrangements. We conclude that the elevation in R loop formation at head-on genes negatively affects DNA replication. To understand the impact of R loops in head-on genes, we tested whether there was a statistical association among mutagenesis, R loops, and the direction of replication-transcription conflict (see below).

### RDHs genome wide promote insertions and deletions

**Fig. 4.**
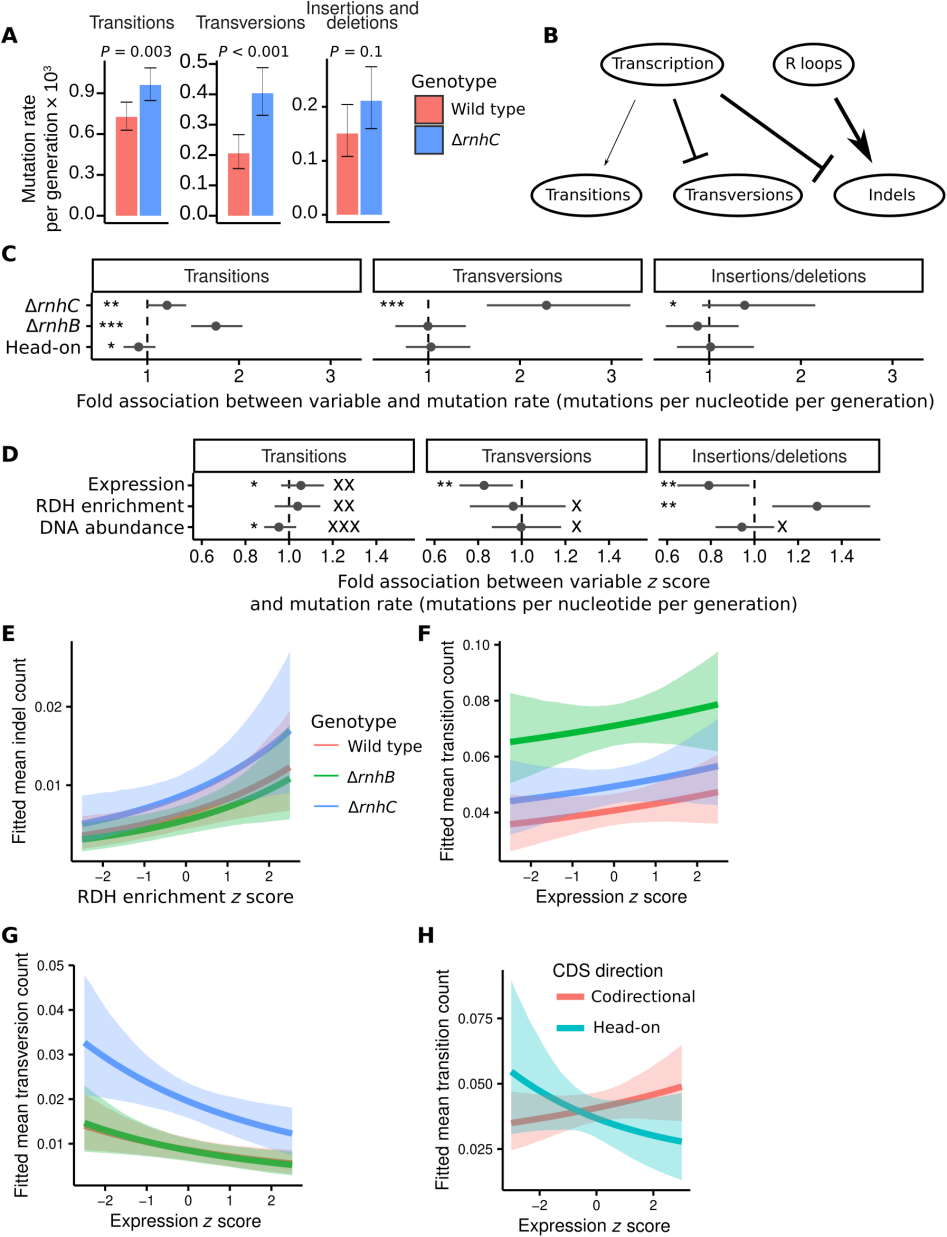
Effects of loss of RnhB and RnhC on mutation rate. (**A**) Bar plots indicate the genome-wide mutation rates from MA line experiments for wild type and Δ*rnhC*. Error bars represent 95% exact Poisson confidence intervals. (**B**) Schematic illustrating the contributions of transcription and R loops to each type of mutation. Line weights are proportional to the median estimate of transcription or R loop association with the indicated mutation. Arrows indicate a positive association, and flat heads denote a negative association. (**C**) Inferred associations between the rate of transition, transversion, or indel occurrence in CDSs and the indicated model parameters for categorical variables. (**D**) Inferred associations between mutation rate in CDSs and the indicated model parameters for continuous variables. All associations for interaction terms are in fig. S4A. (**E**) Plot showing the mean fitted number of indels in CDSs with varying RDH abundance and all other variables held at their mean. The shaded regions indicate the 90% quantile interval for the fitted values. (**F** and **G**) Plots showing the mean fitted number of transitions (F) or transversions (G) in CDSs with varying gene expression and all other variables held at their mean. (**H**) Plot showing the relationship between mean fitted number of transitions in CDSs and gene expression and all other variables held at their mean, stratified by head-on or codirectional. Throughout the figure, *, **, and *** symbols indicate the strength of evidence for a parameter having a substantial association with mutation rate, with * indicating that *K* is from [3,20), ** denoting that *K* is from [20,150), and *** indicating that *K* ≥ 150. Equivalently strong *K*_0_ values are shown with equal numbers of “X” symbols. Note that if a parameter can be very precisely estimated but is close to zero, then it is possible for it to have both high *K* and high *K*_0_.

We have demonstrated above that loss of *rnhC* causes widespread increases in RDH abundance, likely in the form of R loops. We have also shown that SOS induction overcomes severe replication stress near the replication terminus in Δ*rnhC* cells. However, loss of RnhC has led to negligible mutation rate increases (1.3-fold) when mutagenesis is measured using mutation reporter genes (*7*, *20*), and the genome-wide effects of loss of *rnhC* on mutagenesis are unknown. Therefore, to determine the genome-wide outcome of Δ*rnhC* to mutagenesis, we analyzed our MA line data for base pair substitutions and indels. The overall mutation rate for base pair substitutions and small insertions and deletions in Δ*rnhC* was 1.6 × 10^−3^ (data file S2) per generation, which is similar to that for Δ*rnhB* (*10*), and is roughly 50% higher than the wild-type mutation rate of 1.1 × 10^−3^ (*P* < 0.001, rate-ratio test). Analysis of the types of mutations that occurred in Δ*rnhC* cells uncovered increased transition and transversion rates, with no strongly detectable difference in the accumulation of insertions or deletions genome wide (Fig. 4A). The most notable mutagenic effect for base pair substitutions was a twofold increase in transversions (Fig. 4A), a hallmark of the SOS response due to up-regulation of error-prone DNA polymerases, which are more likely to form transversions than replicative enzymes (*32*). Although oxidation of nucleobases can also cause transversions, we found no evidence for induction of the ROS response in Δ*rnhC* cells (table S2). Further, our results showing an increase in transversions are consistent with the constitutive induction of the SOS response we observe in the absence of *rnhC* as described above and the enrichment of transversions in the terminus region where replication forks become laggard and RDHs are enriched (Fig. 3C).

With the diverse types of data we have in hand, we fit a Bayesian statistical model to identify associations between mutation rate in CDSs and several variables, including RDH abundance, expression, and CDS direction relative to replication (head-on versus codirectional). In contrast to the simple rejection of a null hypothesis provided by frequentist methods, our Bayesian approach enabled us to quantify the strength of evidence in favor of each parameter’s value being positive, negative, or nearly equal to zero. We summarize the strength of evidence for each hypothesis in terms of Bayes factors (*K*), for which a high *K* value (>150) indicates very strong evidence, *K* values from 20 to 150 indicate strong evidence, and *K* values from 3 to 20 indicate positive evidence (*33*). In the discussion below, *K* is used to indicate whether a given effect is substantial, whereas *K*_0_ tests the hypothesis that the effect of interest is near zero. Thus, a high *K*_0_ indicates a significant lack of any notable effect. Several aspects of mutagenesis in wild-type, Δ*rnhB*, and Δ*rnhC* MA lines were revealed using this approach (see the “Genome-wide profiling of DNA:RNA hybrids” section in Supplementary Materials and Methods for method and statistical models and see data file S2 for a summary of the inferred parameter estimates). For example, the fit of our statistical model corroborates our conclusion that loss of *rnhC* increases transversion, transition, and indel rates (*K* > 150, 24.6, and 8.6, respectively) (Fig. 4, A and C). In agreement with prior work (*10*), loss of *rnhB* increased CDS transition rate (*K* ≥ 150) due to a previously described error-prone ribonucleotide excision repair (RER) pathway (Fig. 4C) (*10*).

Increasing RDH abundance was associated with increased indel rate (*K* = 59.6) but did not affect transition or transversion rates (*K*_0_ = 68.0 and 4.1, respectively) (Fig. 4, B and D). Although it has been suggested that R loops are particularly mutagenic in head-on genes, our data suggest that, on average, across all native CDSs in the *B. subtilis* genome, associations between mutagenesis and RDH accumulation were independent of gene orientation relative to DNA replication (fig. S4, A and D). We interpret this to indicate that in contrast with engineered mutation reporters designed to detect mutations due to head-on conflict (*7*, *24*), native genes that have evolved in a head-on genomic context have done so, such that the influence of head-on transcription and R loop formation on mutagenesis is minimized. Thus, engineered reporters can demonstrate the underlying molecular mechanisms driving the selective pressures on gene orientation but, in practice selective pressures, have eliminated most potentially damaging conflicts in bacterial genomes.

Increased gene expression has also been associated with increased mutagenesis. We found that gene expression had a very small positive association with transition rate (*K* = 5.1, *K*_0_ = 46.6), whereas transversion and indel rates were lower as expression increased (*K* = 38.2 and 23.1, respectively) (Fig. 4, B, C, F, and G). In addition, for head-on genes, our data show the opposite association between transition rate and gene expression. Overall, we show that as expression of head-on genes increases, their transition rate decreases (Fig. 4G; fig. S4, A and C; and Discussion). We conclude that loss of *rnhC* results in an increase in transition, transversion, and indel mutations, while loss of *rnhB* yields an increase in transitions. Further, we conclude that R loop–dependent mutagenesis in Δ*rnhC* is independent of gene orientation and is instead dependent on the extent to which RDHs accumulate and the length of a genomic locus over which they accumulate.

We demonstrated above that transcription and RDH accumulation are correlated (Fig. 2B); therefore, we tested whether the correlation between gene expression and RDH accumulation affected our ability to separately infer the association between each variable and mutation rates by fitting two additional statistical models. Each was identical to the model described above, with the exception that the first additional model lacked any terms associated with gene expression and the second lacked any terms associated with RDH enrichment. If either variable affected inferences about the other’s association with mutation rate, then this approach would have detected that relationship. However, the inferred associations between expression or HBD accumulation and mutation rate did not change appreciably when leaving the other variable out of a given model, and the predictive performance as measured by leave-one-out cross-validation (LOO-CV) was not influenced by leaving either variable out of the model (see the “Bayesian inference of parameter association with mutation rate” section in Supplementary Materials and Methods and table S3 for details). Therefore, although expression is correlated with RDH abundance, each influences mutagenesis separately. Knowing both expression level and RDH abundance will provide a much better prediction of the gene’s mutation rate than knowing either parameter in isolation.

## DISCUSSION

RDHs arise in many different forms and have been shown to affect replication fork progression, mutation rate, and cell survival (*7*, *10*, *20*, *21*, *34*). Here, we investigate the consequences of loss of either *rnhB* or *rnhC* in vivo to RDH accumulation, replication fork progression, and genome-wide mutation rates. The major finding of our work is that R loops increase indel rates, whereas loss of *rnhC* leads to increased transversion rates due to R loop–induced replication stress and induction of the SOS response. We are now able to propose models for the roles of RnhB and RnhC in RDH removal and their consequences for genome stability in bacteria.

Loss of *rnhB* from *B. subtilis* cells causes increased transition mutations and alterations to the genomic distribution of RDHs without causing increased sensitivity to DNA damaging agents. The analysis of RDH pulldowns from cells lacking *rnhB* showed an increase in RDH abundance in UTRs and in the first 100 bp of CDSs. However, we demonstrate that purified RnhB shows little activity on an R loop lacking a covalent RNA-DNA junction in vitro, while RnhC shows robust activity. The mechanisms underlying the stabilization of RDHs in Δ*rnhB* strongly suggest formation of an intermediate that contains a covalent RNA-DNA junction, the chemical structure recognized by RnhB (*19*). While it is possible to knock out both *rnhB* and *rnhC* in a single *B. subtilis* strain, Δ*rnhB*Δ*rnhC* cells have a slow growth rate (*20*). We have also found that each time we sequence the genome of a Δ*rnhB*Δ*rnhC* isolate, we identify compensatory mutations (*35*), causing us to abandon further work on these strains. Together, we suggest that the major benefits of R loop digestion by RnhC are to limit replication stress and fork reversal [Figs. 3 and 4 and (*27*)] and to keep 3′-termini of R loops from being used inappropriately as primers for DNA synthesis. When transcripts are used to prime DNA synthesis, RnhB can act on such inappropriate priming events if R loops escape the activity of RnhC or if cells lack *rnhC* entirely (*19*, *35*). We propose that increased RDH abundance at 5′-UTRs and early in CDSs in Δ*rnhB* cells represents the fraction of R loops that escape RnhC action and are used to prime DNA synthesis at sites of replication fork blockage. These priming events generate a covalent RNA-DNA junction leading to resolution by RnhB. In the absence of *rnhB* the repriming events are stabilized and detected in our pull-down assay. This model would also explain how RDHs in Δ*rnhB* cells show little dependence on transcription.

We show that Δ*rnhC* cells are exquisitely sensitive to DNA damage [Fig. 3F, fig. S3B, and (*19*)] and accumulate RDHs in both gene orientations in a transcription-dependent manner. We considered the possibility that the Δ*rnhC* growth inhibition in response to DNA damage was due to failed primer removal from Okazaki fragments, which was compounded by the addition of exogenous DNA damage. If this had been the case, then overexpression of RnhB or Pol I would have provided moderate rescue, as RnhB and Pol I are involved in RER and Okazaki fragment processing in *B. subtilis* (*10*, *20*, *35*). However, neither *rnhB* nor Pol I (*polA*) overexpression rescued any sensitivity, suggesting that the sensitivities were primarily due to deficiencies in R loop resolution, an activity in which *B. subtilis* RnhC has been implicated [Fig. 1, A and B, and (*7*)]. We find it likely that the SOS response is nearly saturated in Δ*rnhC* cells, which would cause both their sensitivity to DNA damaging agents and the synergistic sensitivity of Δ*rnhC lexA[G92D]* cells to those same agents.

In addition to DNA damaging agents, Δ*rnhC* cells are sensitive to osmotic stress, cold temperatures, lysozyme, oxidative stress, and HU (*7*, *19*, *35*). One model suggests that the multiple sensitivities of Δ*rnhC* cells are caused by impaired induction of head-on stress-induced genes (*7*). Another possible explanation arising from the work presented here is that persistent, genome-wide R loop formation in all genes, independent of orientation (Fig. 2, B and C), causes DNA replication stress that is further exacerbated by an exogenously applied stress condition.

While prior work in *B. subtilis* has suggested that *rnhC* overexpression may decrease mutagenesis in head-on reversion reporter genes (*7*), studies in yeast using head-on and codirectional reporter genes have shown that replication-transcription conflicts caused by R loops are independent of gene orientation (*36*). Our approach has the advantage of querying genome-wide mutagenesis and RDHs in native gene contexts. Understanding the effect in native gene context is important because native genes have already evolved under selective pressure from effects of gene position and orientation on mutagenic potential. Our work shows that RDHs mainly increase indel rates in coding sequences and promote transversions in Δ*rnhC* cells where replication forks encounter R loop impediments. Oxidation of nucleobases can also cause transversions, but RNA-seq failed to show induction of the ROS response in Δ*rnhC* cells (table S2). An important feature of our finding is that the transversion mutations identified in *B. subtilis* are locus specific, with mutations occurring in the *pps* operon located in the chromosomal terminus.

The consensus view of the association of gene expression with mutagenesis is that as gene expression increases, so does mutagenesis (*37*–*41*), and while our inference corroborates prior evidence that increased transcription increases the transition mutation rate of a gene, we found that the effect is likely to be very small in the genes natively found in *B. subtilis*. The stronger effects of transcription on mutagenesis are the negative associations between expression and transversion and indel rates. We assert this negative effect of transcription on transversion and indel rates is likely due to transcription-coupled nucleotide excision repair acting on the types of DNA damage that would otherwise cause transversions and indels [reviewed in (*42*, *43*)].

The types of mutations caused by, and the evolutionary consequences of, head-on replication-transcription conflict and conflict between DNA replication and R loops in head-on genes are a subject of ongoing discussion (*7*, *24*, *31*, *36*, *44*, *45*). Bacterial genes are enriched in the codirectional orientation because of negative selection against head-on conflicts, which disrupt DNA replication and cause mutagenesis (*46*). Therefore, head-on genes tend to be those that are either more tolerant to mutation or produce fewer conflicts [reviewed in (*46*, *47*)], and the few native head-on genes that do produce substantial conflicts will likely be purified from the genome over time. By contrast, synthetic mutation reporter genes have not been subject to the evolutionary pressures shaping the genome (*7*, *24*) and are able to provide insights into how mutagenesis is affected by extreme engineered conflicts that select for specific mutations. Engineered gene conflict reporters are thus best used to illustrate the mechanisms underlying the selective pressures that have acted to purify or minimize head-on conflicts in native genes. Although the results presented here and elsewhere (*48*) do not demonstrate a general association between gene direction and mutagenesis, we have identified one native locus, *ppsABDE*, that produces results similar to those from engineered mutation reporters. Without *rnhC*, *ppsABDE* accumulates transversions and structural variants.

The *pps* locus also exhibits substantial replication stress in Δ*rnhC* cells, and the increased transversion rate can be attributed to induction of the SOS response, which we show is required for replication to proceed through *ppsABDE* in Δ*rnhC* cells. Among the multiple pathways toward mutagenesis, we find two major pathways to be the most likely candidates for mutagenesis at *ppsABDE* in Δ*rnhC* cells. Because SOS induction includes the up-regulation of error-prone Y-family polymerases and the essential error-prone replicative DNA polymerase DnaE [reviewed in (*32*)], increased transversions and detrimental structural variants near the terminus suggest that SOS induction and error-prone DNA polymerases are likely to have greater involvement in DNA replication at this chromosomal location in Δ*rnhC* cells. Whatever the ultimate mechanistic cause of mutagenesis near *ppsABDE*, when specific conditions are met, including head-on orientation, stable R loop formation, considerable length, SOS induction, and location late in the replichore, one native locus in *B. subtilis* shows increased mutagenesis in the head-on orientation. The mutations arising in the *pps* operon are unlikely to provide an adaptive benefit as they are often large deletions.

Together, our whole-genome data allow reconciliation and mechanistic explanation of a wide range of phenomena related to the interplay of RDH formation, genome organization, gene orientation, and mutagenesis that have been observed in different experimental systems. Global analysis of mutagenic profiles demonstrates that regardless of their origin, RDHs are associated with increased insertion/deletion mutations. While loss of function of either *rnhB* or *rnhC* causes increases in RDH accumulation and increases in overall mutation rates, the differing effects of loss of the two genes arise due to resolution of different types of RDHs, resulting in different types of mutations (transitions versus transversions and indels) and different biases in where RDHs accumulate. For naturally occurring genes under baseline physiological conditions, a head-on orientation relative to transcription is not mutagenic, but loss of *rnhC* function unmasks the mutagenic potential of very specific head-on oriented genes when R loop formation is increased. In general, it appears that the *B. subtilis* genome has reached a point of equilibrium with respect to gene orientation, in which regularly expressed genes in the head-on orientation have undergone selective pressure to minimize R loop formation and consequently mutagenesis. These findings explain the discrepancy between mutagenesis rates observed in long, highly expressed artificial reporters versus native genes.

## MATERIALS AND METHODS

### Bacteriology

All strains used in this study are described in data file S1 in the Supplementary Materials. Cells were grown in S7_50_-defined minimal medium (*49*) or LB medium at 30°C. For antibiotics, cells with *recA-gfp* were selected for using spectinomycin (100 μg/ml), and erythromycin (5 μg/ml). Further details are given in the “Bacteriology and cloning” section in Supplementary Materials and Methods.

### Spot titer assays

Assays were performed essentially as described in (*19*). Briefly, a single colony of the indicated strain was used to inoculate 3 ml of LB media and grown to an optical density at 600 (OD_600_) between 1 and 1.5. Cultures were then normalized to an OD_600_ = 1 in a 0.85% saline solution and serial diluted to 10^−5^. A total volume of 5 μl was spotted for each dilution on LB agar containing the indicated concentrations of mitomycin C, methyl methanesulfonate, phleomycin, HU, and or isopropyl-β-d-thiogalactopyranoside. Plates were then incubated overnight at 30°C and imaged the following morning. Concentrations of DNA damaging agents used were screened to identify the lowest concentration that affected the mutant growth while having little effect on wild-type cells.

### Fluorescence microscopy

RecA–green fluorescent protein (GFP) and TagC-GFP strains were imaged as described previously (*50*, *51*). See the “Fluorescence microscopy” section in Supplementary Materials and Methods for further information.

### MA lines

Procedures were performed as described (*10*, *31*). Previously published wild-type (*B. subtilis* PY79) and Δ*rnhB* MA line data were compiled (*10*, *31*). Structural variation in MA lines were detected using the software “breakdancer” (*52*). Structural variants detected within 100 bp of the intended genetic alteration for an MA line were removed from further analysis. For instance, *rnhB* has the start and end positions 1,640,489 and 1,641,256, respectively, so structural variants detected by breakdancer within the location from genomic coordinates 1,640,389 to 1,641,356 were removed from this study. Upon inspection of the structural variants in our MA lines, we noticed that a Δ*rnhC* line contained a deletion of the 3′ end of the *mutS* gene. Because *mutS* is required for DNA mismatch repair, the mutation rate and spectrum would be strongly affected (*31*) by this structural variation in *mutS*. Therefore, we removed the line from this study.

### Plotting transversion density

To calculate and plot the transversion densities used in Fig. 3, we used the geom_line function from the R package ggplot2, with the arguments stat = “density” and adjust = 0.2.

### Genome-wide profiling of RDHs

Details of the preparation of epitope-tagged hybrid binding domains, cell growth for pull-down experiments, sequencing library preparation, and subsequent data analysis are given in the “Genome-wide profiling of DNA:RNA hybrids” section in Supplementary Materials and Methods. Two biological replicates were performed for HBD pulldowns.

#### Cell lysis and nucleic acid extraction

RDH pull-down experiments were performed by first extracting total nucleic acid from snap-frozen cells of the appropriate genotypes. Equivalent *C. crescentus* spike-in pellets (1 per sample) were resuspended in 1× low salt lysis buffer [660 μl per pellet; 10 mM tris-HCl (pH 8.0) and 50 mM NaCl], transferred to a single *B. subtilis* pellet, resuspended via pipetting, and sonicated four times for 5 s at 25% amplitude with a 15-s pause between pulses (Branson digital sonifier). Then, 1/10 volume of 10% SDS and 10 μl of proteinase K (50 mg/mL; EO0491, Thermo Fisher Scientific) were added to each sample, mixed, and then incubated 55°C for 5 min. Then, 1/10 volume of 3 M sodium acetate and 500 μl of phenol/chloroform/isoamyl alcohol were mixed in each tube. Tubes were incubated with mixing (600 rpm) for 6 min at 65°C. After sitting on ice for 5 min, the layers were separated via centrifugation (10 min, 16,000*g*, 4°C). The water layer was transferred to a separate tube containing 500 μl of chloroform, vortexed, and then spun for 5 min at 16,000*g* at 4°C. The aqueous layer was transferred to a separate 2-ml microfuge tube, and ≥2.5 volumes of ice-cold, 100% ethanol were added. Tubes were incubated −20°C for 60 min. Nucleic acids were pelleted by centrifugation (15 min, 16,000*g*, 4°C), the supernatant was removed, and the pellet was washed once with 80% ethanol and then air dried until ethanol was absent. Nucleic acids were dissolved in 10 mM tris (pH 7.5). This solution was stored at −80°C until the following step.

#### Digestion of nucleic acids for RNA:DNA extraction from cells

To each tube/sample, 20 μl of 10× micrococcal nuclease (MNase) buffer (New England Biolabs Inc., Ipswich MA, USA), 0.5 μl of 1 M MgCl_2_, 2 μl of RNase inhibitor (M0314, New England Biolabs Inc., Ipswich MA, USA), and 2 μl of MNase (M0247, New England Biolabs Inc., Ipswich MA, USA) were mixed in and tubes were promptly placed on ice. After 20 min, 10 μl of 500 mM EGTA was added to each sample. The concentration of nucleic acid for each sample was determined by measurement of *A*_260_ (absorbance at 260 nm) (1 to 100 dilution). Aliquots of 500 μg were made of each sample. Then, 1/10,000 of the sample, 50 ng of previously folded RNA-DNA hair-pin spike-in (P2918; data file S1) was added to each sample. For most of these samples, 1X PBST [137 mM NaCl, 12 mM phosphate, 2.7 mM KCl (pH 7.4), and 0.2% Tween 20] was added to achieve a 200-μl volume and mixed by vortex. Then, 0.5 μl of 1 MgCl_2_ and 2 μl of RNase inhibitor (NEB) were added to each sample and mixed by vortex. A 20 μl of aliquot per sample was placed in a separate tube as an input sample and then stored at −80°C. The remaining digested nucleic acids were used directly for HBD-mediated pulldown as described below.

#### HBD-mediated pulldown of extracted and digested RDHs

Freshly thawed on ice, purified hemagglutinin (HA)–tagged HBD protein was added to the remaining nucleic acid digestion from each sample and mixed by repeated inversion. The HBD proteins were allowed to bind to the digested nucleic acids for 20 min at room temperature. Magnetic anti-HA beads (25 μl per tube; Pierce) were washed twice briefly with 1× PBST [137 mM NaCl, 12 mM phosphate, 2.7 mM KCl (pH 7.4), and 0.2% Tween 20], and the liquid removed and replaced with the remaining nucleic acid digestion. Tubes were incubated at room temperature on the undulating shaker for 20 min. Unbound material was removed. Beads were washed with 200 μl of 1× PBST and with 200 μl of 1× PBST + 850 mM NaCl_2_, then resuspended in 100 μl of 1× PBST, and moved to a separate tube. After the 1× PBST was removed, beads were resuspended in 50 μl of 1× TE [10 mM Tris (pH 8), 1 mM EDTA] and incubated at 95°C for 5 min. Samples were returned to a magnetic stand, allowed to clear, and liquid transferred to DNA LoBind tubes (Eppendorf). Samples were stored at −80°C.

#### Next-generation sequencing and data analysis

Illumina sequencing libraries were prepared from the extracted and input samples (see the “Genome-wide profiling of DNA:RNA hybrids” section in Supplementary Materials and Methods) and sequenced on an Illumina NextSeq 500 instrument using 39 × 38–bp paired end reads. Data were subsequently processed using an in-house pipeline in which read alignments were processed to yield extracted:input ratios at each position on the chromosome, followed by normalization to robust *z* scores. Details are given in the “Genome-wide profiling of DNA:RNA hybrids” section in Supplementary Materials and Methods

### Statistical modeling of mutagenesis

A detailed description of the model used to infer parameter estimates for mutation rate can be found in the “Bayesian inference of parameter association with mutation rate” section in Supplementary Materials and Methods.

### Purification of RnhB and RnhC for in vitro characterization

All RNase H proteins were purified and assayed as described previously (*10*) . The R loop digestion buffer used 4 nM RnhC, 50 nM RnhB, or 50 nM RnhC catalytic mutant in the following reaction buffer: 20 μl of RNase H buffer [10 mM tris-HCl (pH 8), 50 mM NaCl, 1 mM MgCl_2_, or 10 μM MnCl_2_]. For in vivo relevant metal concentrations, both 1 mM MgCl_2_ and 10 μM MnCl_2_ were used as described previously (*19*).

### R loop substrate formation and RNase H assay

The R loop substrate was based on a previously published R loop substrate (*53*). The R loop was assembled by first annealing 10 μM oJR336 and oJR335 in a 50-μl solution of annealing buffer [10 mM tris-HCl (pH 8) and 50 mM NaCl) by heating to 80°C and allowed cooling to 25°C. Then, 10 μM oJR332 was added and the solution was heated to 40°C and again allowed cooling to 25°C. The product was then visualized by native–polyacrylamide gel electrophoresis in an 8% gel, and the R loop substrate was gel-extracted and diluted to 0.5 μM final concentration. Gel extraction and verification of the substrate are described in detail in the Supplementary Materials.

## Supplementary Materials

### This PDF file includes:

Supplemental Materials and Methods Figs. S1 to S4 Table S1 to S3 Legends for data files S1 and S2 References
